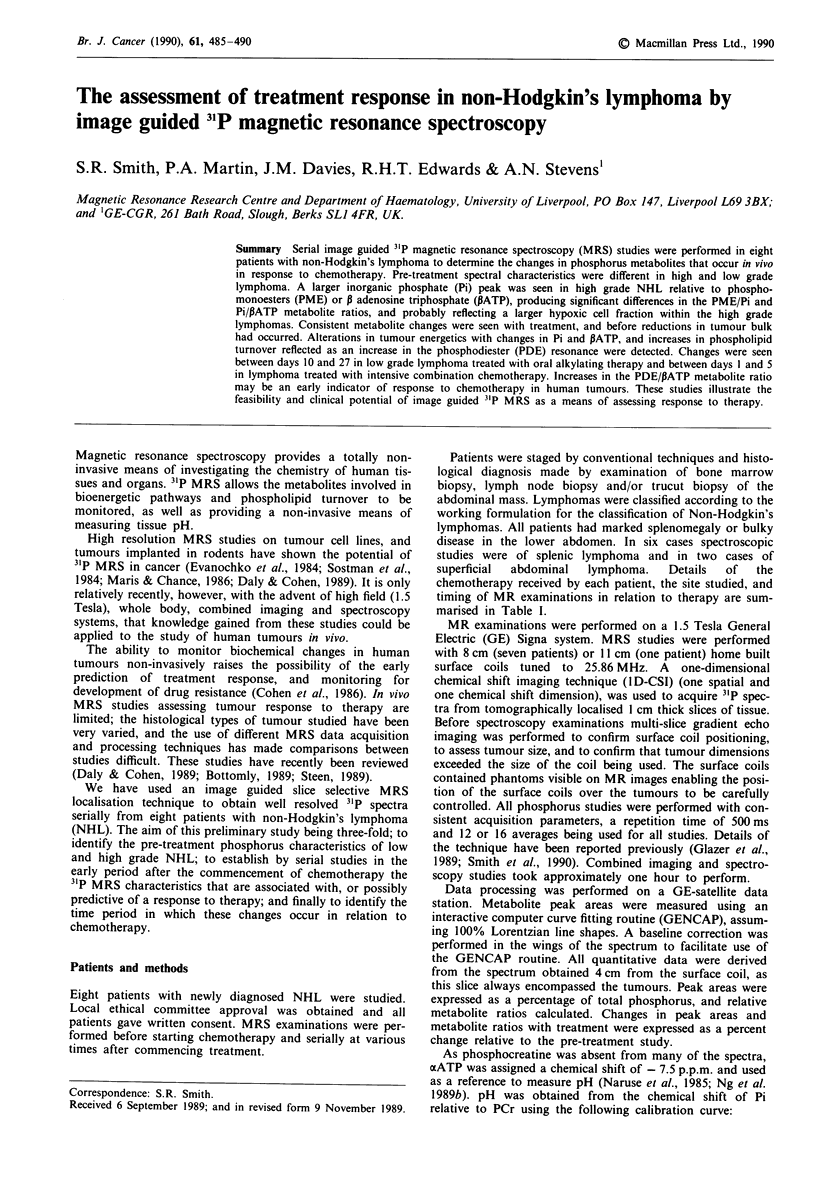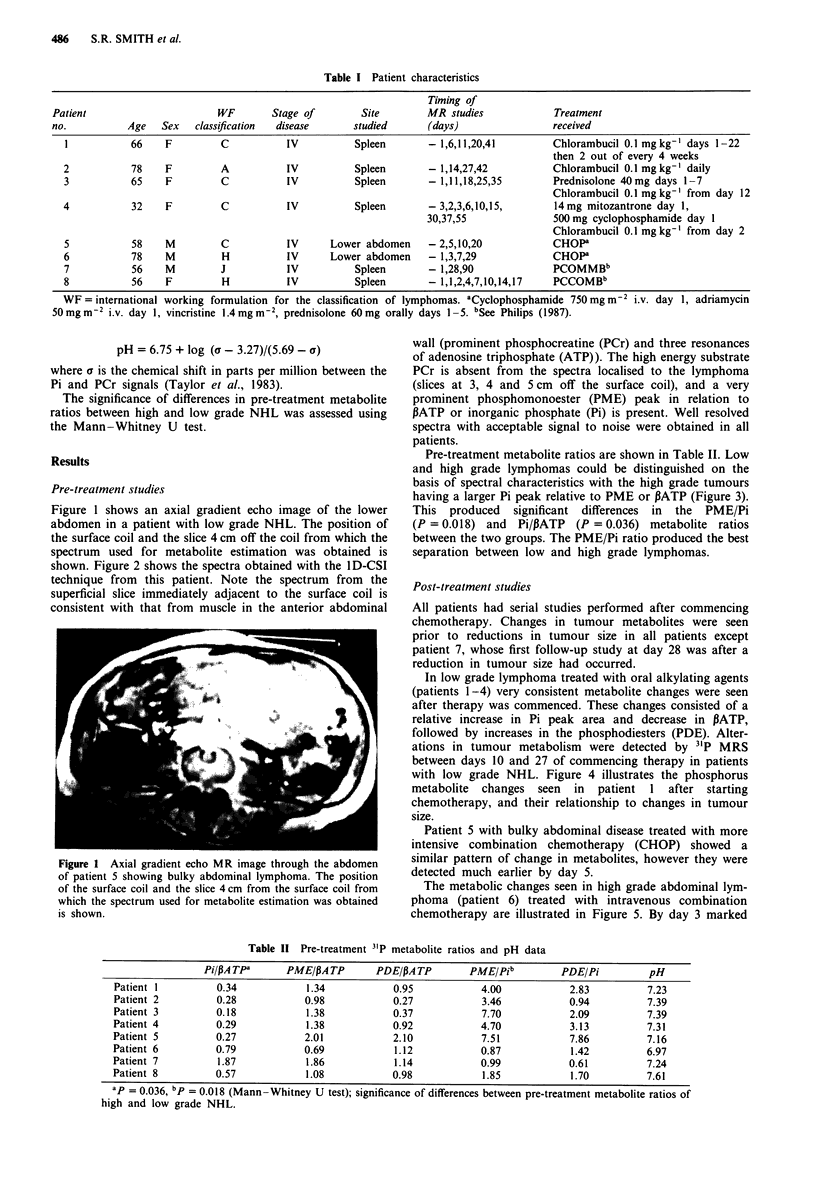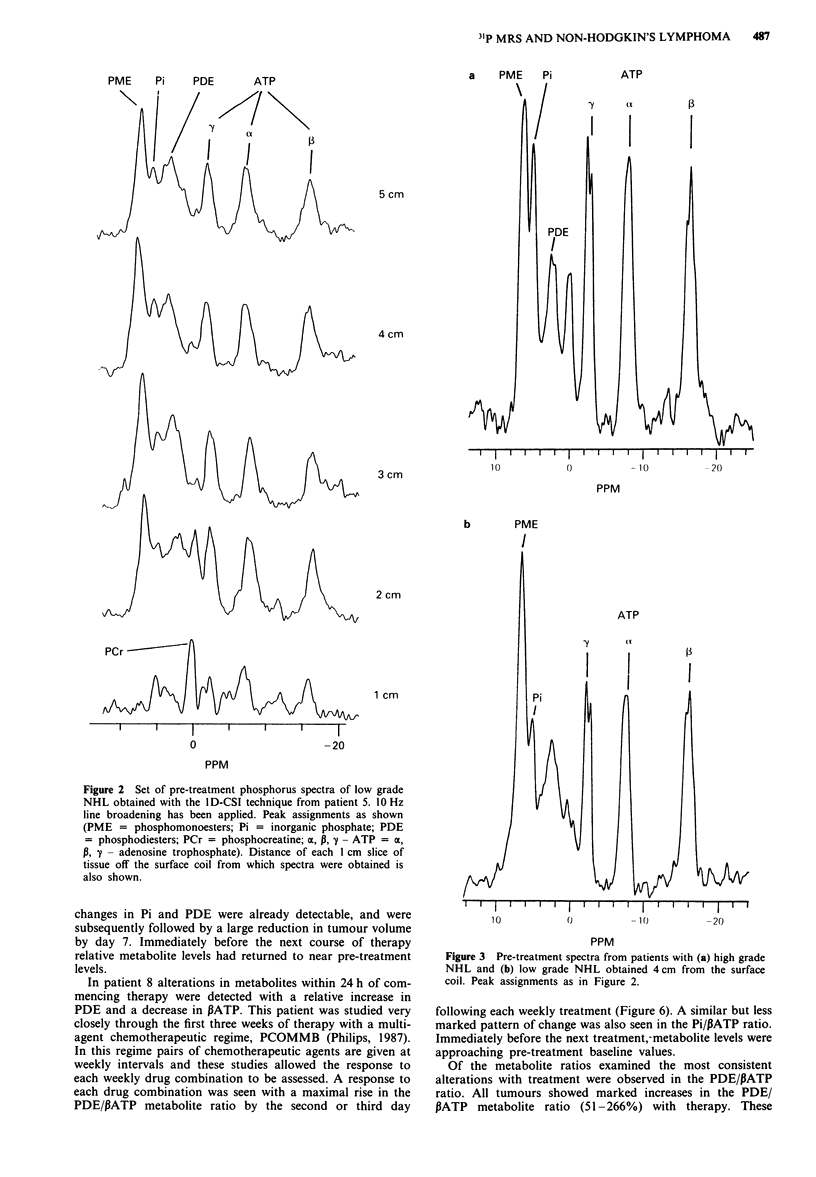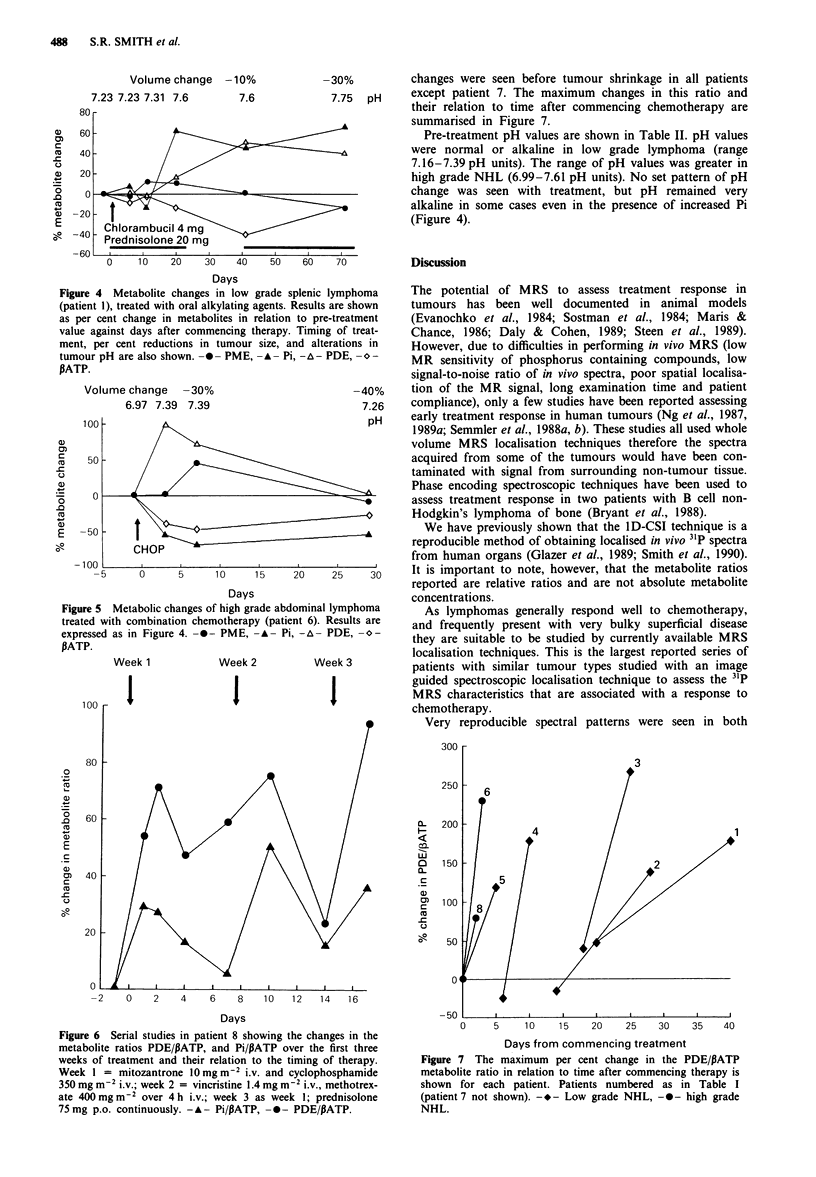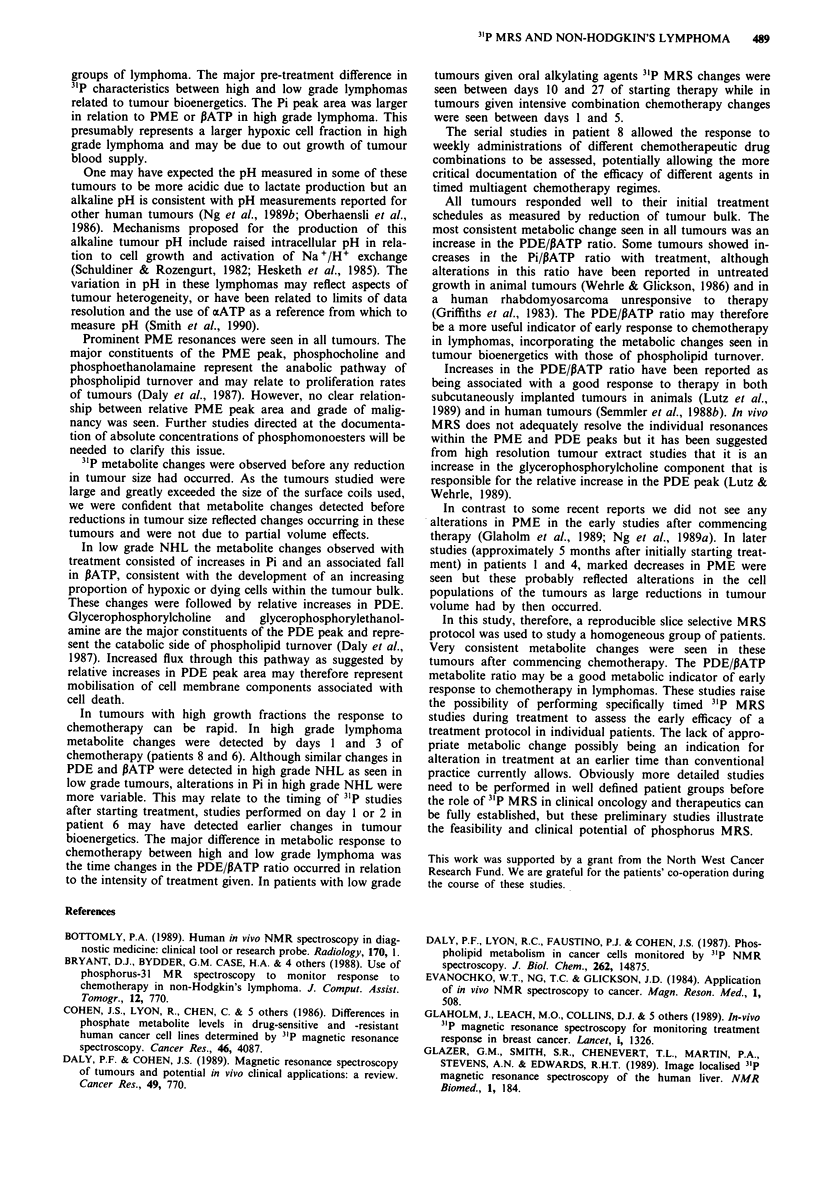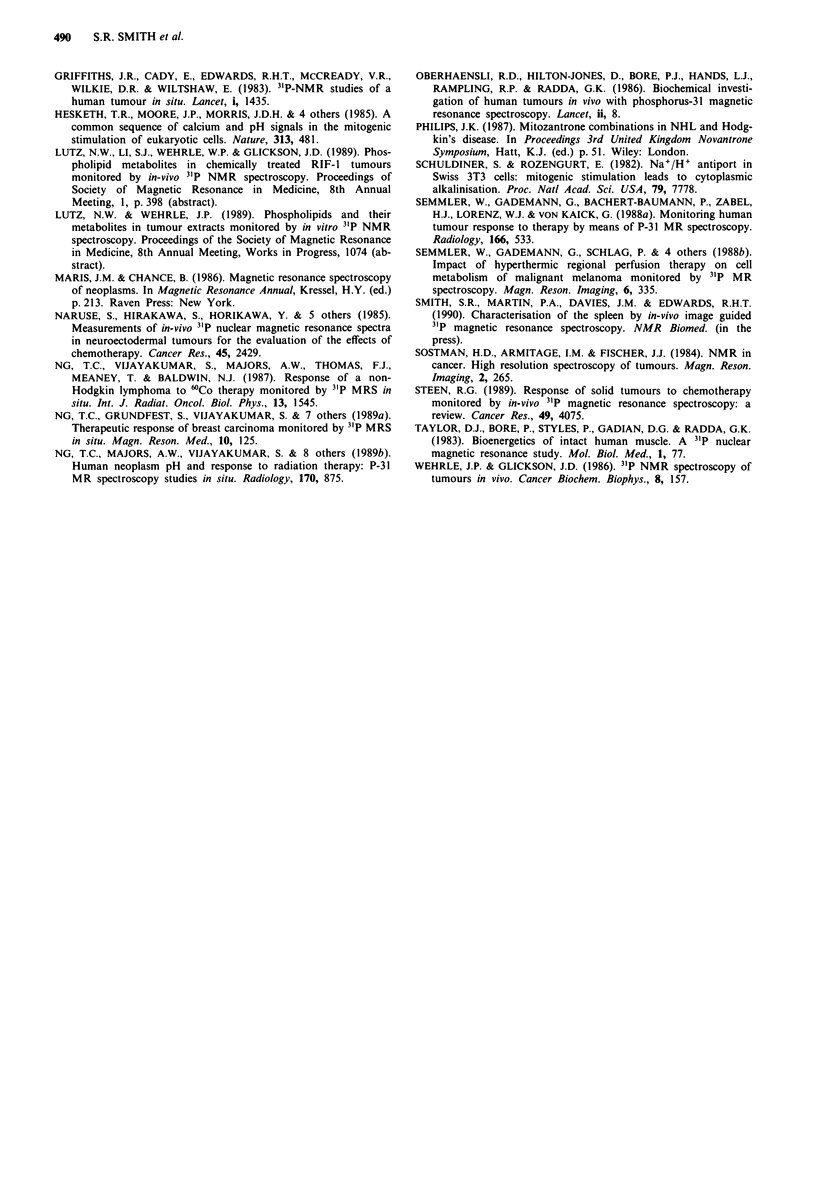# The assessment of treatment response in non-Hodgkin's lymphoma by image guided 31P magnetic resonance spectroscopy.

**DOI:** 10.1038/bjc.1990.107

**Published:** 1990-03

**Authors:** S. R. Smith, P. A. Martin, J. M. Davies, R. H. Edwards, A. N. Stevens

**Affiliations:** Magnetic Resonance Research Centre, University of Liverpool, UK.

## Abstract

**Images:**


*Br. J. Cancer* (1990), **61**, 485–490 © Macmillan Press Ltd., 1990

### The assessment of treatment response in non-Hodgkin's lymphoma by image guided $^{31}$P magnetic resonance spectroscopy

S.R. Smith, P.A. Martin, J.M. Davies, R.H.T. Edwards & A.N. Stevens[1]

*Magnetic Resonance Research Centre and Department of Haematology, University of Liverpool, PO Box 147, Liverpool L69 3BX; and [1]GE-CGR, 261 Bath Road, Slough, Berks SL1 4FR, UK.*

**Summary** Serial image guided $^{31}$P magnetic resonance spectroscopy (MRS) studies were performed in eight patients with non-Hodgkin's lymphoma to determine the changes in phosphorus metabolites that occur *in vivo* in response to chemotherapy. Pre-treatment spectral characteristics were different in high and low grade lymphoma. A larger inorganic phosphate (Pi) peak was seen in high grade NHL relative to phosphomonoesters (PME) or β adenosine triphosphate (βATP), producing significant differences in the PME/Pi and Pi/βATP metabolite ratios, and probably reflecting a larger hypoxic cell fraction within the high grade lymphomas. Consistent metabolite changes were seen with treatment, and before reductions in tumour bulk had occurred. Alterations in tumour energetics with changes in Pi and βATP, and increases in phospholipid turnover reflected as an increase in the phosphodiester (PDE) resonance were detected. Changes were seen between days 10 and 27 in low grade lymphoma treated with oral alkylating therapy and between days 1 and 5 in lymphoma treated with intensive combination chemotherapy. Increases in the PDE/βATP metabolite ratio may be an early indicator of response to chemotherapy in human tumours. These studies illustrate the feasibility and clinical potential of image guided $^{31}$P MRS as a means of assessing response to therapy.

Magnetic resonance spectroscopy provides a totally non-invasive means of investigating the chemistry of human tissues and organs. $^{31}$P MRS allows the metabolites involved in bioenergetic pathways and phospholipid turnover to be monitored, as well as providing a non-invasive means of measuring tissue pH.

High resolution MRS studies on tumour cell lines, and tumours implanted in rodents have shown the potential of $^{31}$P MRS in cancer (Evanochko *et al.*, 1984; Sostman *et al.*, 1984; Maris & Chance, 1986; Daly & Cohen, 1989). It is only relatively recently, however, with the advent of high field (1.5 Tesla), whole body, combined imaging and spectroscopy systems, that knowledge gained from these studies could be applied to the study of human tumours *in vivo*.

The ability to monitor biochemical changes in human tumours non-invasively raises the possibility of the early prediction of treatment response, and monitoring for development of drug resistance (Cohen *et al.*, 1986). *In vivo* MRS studies assessing tumour response to therapy are limited; the histological types of tumour studied have been very varied, and the use of different MRS data acquisition and processing techniques has made comparisons between studies difficult. These studies have recently been reviewed (Daly & Cohen, 1989; Bottomly, 1989; Steen, 1989).

We have used an image guided slice selective MRS localisation technique to obtain well resolved $^{31}$P spectra serially from eight patients with non-Hodgkin's lymphoma (NHL). The aim of this preliminary study being three-fold; to identify the pre-treatment phosphorus characteristics of low and high grade NHL; to establish by serial studies in the early period after the commencement of chemotherapy the $^{31}$P MRS characteristics that are associated with, or possibly predictive of a response to therapy; and finally to identify the time period in which these changes occur in relation to chemotherapy.

### Patients and methods

Eight patients with newly diagnosed NHL were studied. Local ethical committee approval was obtained and all patients gave written consent. MRS examinations were performed before starting chemotherapy and serially at various times after commencing treatment.

Correspondence: S.R. Smith.
Received 6 September 1989; and in revised form 9 November 1989.

Patients were staged by conventional techniques and histological diagnosis made by examination of bone marrow biopsy, lymph node biopsy and/or trucut biopsy of the abdominal mass. Lymphomas were classified according to the working formulation for the classification of Non-Hodgkin's lymphomas. All patients had marked splenomegaly or bulky disease in the lower abdomen. In six cases spectroscopic studies were of splenic lymphoma and in two cases of superficial abdominal lymphoma. Details of the chemotherapy received by each patient, the site studied, and timing of MR examinations in relation to therapy are summarised in Table I.

MR examinations were performed on a 1.5 Tesla General Electric (GE) Signa system. MRS studies were performed with 8 cm (seven patients) or 11 cm (one patient) home built surface coils tuned to 25.86 MHz. A one-dimensional chemical shift imaging technique (1D-CSI) (one spatial and one chemical shift dimension), was used to acquire $^{31}$P spectra from tomographically localised 1 cm thick slices of tissue. Before spectroscopy examinations multi-slice gradient echo imaging was performed to confirm surface coil positioning, to assess tumour size, and to confirm that tumour dimensions exceeded the size of the coil being used. The surface coils contained phantoms visible on MR images enabling the position of the surface coils over the tumours to be carefully controlled. All phosphorus studies were performed with consistent acquisition parameters, a repetition time of 500 ms and 12 or 16 averages being used for all studies. Details of the technique have been reported previously (Glazer *et al.*, 1989; Smith *et al.*, 1990). Combined imaging and spectroscopy studies took approximately one hour to perform.

Data processing was performed on a GE-satellite data station. Metabolite peak areas were measured using an interactive computer curve fitting routine (GENCAP), assuming 100% Lorentzian line shapes. A baseline correction was performed in the wings of the spectrum to facilitate use of the GENCAP routine. All quantitative data were derived from the spectrum obtained 4 cm from the surface coil, as this slice always encompassed the tumours. Peak areas were expressed as a percentage of total phosphorus, and relative metabolite ratios calculated. Changes in peak areas and metabolite ratios with treatment were expressed as a percent change relative to the pre-treatment study.

As phosphocreatine was absent from many of the spectra, αATP was assigned a chemical shift of − 7.5 p.p.m. and used as a reference to measure pH (Naruse *et al.*, 1985; Ng *et al.* 1989*b*). pH was obtained from the chemical shift of Pi relative to PCr using the following calibration curve:

**Table I** Patient characteristics

| Patient no. | Age | Sex | WF classification | Stage of disease | Site studied | Timing of MR studies (days) | Treatment received |
|---|---|---|---|---|---|---|---|
| 1 | 66 | F | C | IV | Spleen | – 1,6,11,20,41 | Chlorambucil 0.1 mg $kg^{-1}$ days 1–22 then 2 out of every 4 weeks |
| 2 | 78 | F | A | IV | Spleen | – 1,14,27,42 | Chlorambucil 0.1 mg $kg^{-1}$ daily |
| 3 | 65 | F | C | IV | Spleen | – 1,11,18,25,35 | Prednisolone 40 mg days 1–7 Chlorambucil 0.1 mg $kg^{-1}$ from day 12 |
| 4 | 32 | F | C | IV | Spleen | – 3,2,3,6,10,15, 30,37,55 | 14 mg mitozantrone day 1, 500 mg cyclophosphamide day 1 Chlorambucil 0.1 mg $kg^{-1}$ from day 2 |
| 5 | 58 | M | C | IV | Lower abdomen | – 2,5,10,20 | CHOP[a] |
| 6 | 78 | M | H | IV | Lower abdomen | – 1,3,7,29 | CHOP[a] |
| 7 | 56 | M | J | IV | Spleen | – 1,28,90 | PCOMMB[b] |
| 8 | 56 | F | H | IV | Spleen | – 1,1,2,4,7,10,14,17 | PCCOMB[b] |

WF = international working formulation for the classification of lymphomas. [a]Cyclophosphamide 750 mg $m^{-2}$ i.v. day 1, adriamycin 50 mg $m^{-2}$ i.v. day 1, vincristine 1.4 mg $m^{-2}$, prednisolone 60 mg orally days 1–5. [b]See Philips (1987).

$$pH = 6.75 + \log (\sigma - 3.27)/(5.69 - \sigma)$$

where $\sigma$ is the chemical shift in parts per million between the Pi and PCr signals (Taylor *et al.*, 1983).

The significance of differences in pre-treatment metabolite ratios between high and low grade NHL was assessed using the Mann–Whitney U test.

### Results

### *Pre-treatment studies*

Figure 1 shows an axial gradient echo image of the lower abdomen in a patient with low grade NHL. The position of the surface coil and the slice 4 cm off the coil from which the spectrum used for metabolite estimation was obtained is shown. Figure 2 shows the spectra obtained with the 1D-CSI technique from this patient. Note the spectrum from the superficial slice immediately adjacent to the surface coil is consistent with that from muscle in the anterior abdominal wall (prominent phosphocreatine (PCr) and three resonances of adenosine triphosphate (ATP)). The high energy substrate PCr is absent from the spectra localised to the lymphoma (slices at 3, 4 and 5 cm off the surface coil), and a very prominent phosphomonoester (PME) peak in relation to βATP or inorganic phosphate (Pi) is present. Well resolved spectra with acceptable signal to noise were obtained in all patients.

**Figure 1** Axial gradient echo MR image through the abdomen of patient 5 showing bulky abdominal lymphoma. The position of the surface coil and the slice 4 cm from the surface coil from which the spectrum used for metabolite estimation was obtained is shown.

Pre-treatment metabolite ratios are shown in Table II. Low and high grade lymphomas could be distinguished on the basis of spectral characteristics with the high grade tumours having a larger Pi peak relative to PME or βATP (Figure 3). This produced significant differences in the PME/Pi ($P = 0.018$) and Pi/βATP ($P = 0.036$) metabolite ratios between the two groups. The PME/Pi ratio produced the best separation between low and high grade lymphomas.

### *Post-treatment studies*

All patients had serial studies performed after commencing chemotherapy. Changes in tumour metabolites were seen prior to reductions in tumour size in all patients except patient 7, whose first follow-up study at day 28 was after a reduction in tumour size had occurred.

In low grade lymphoma treated with oral alkylating agents (patients 1–4) very consistent metabolite changes were seen after therapy was commenced. These changes consisted of a relative increase in Pi peak area and decrease in βATP, followed by increases in the phosphodiesters (PDE). Alterations in tumour metabolism were detected by $^{31}$P MRS between days 10 and 27 of commencing therapy in patients with low grade NHL. Figure 4 illustrates the phosphorus metabolite changes seen in patient 1 after starting chemotherapy, and their relationship to changes in tumour size.

Patient 5 with bulky abdominal disease treated with more intensive combination chemotherapy (CHOP) showed a similar pattern of change in metabolites, however they were detected much earlier by day 5.

The metabolic changes seen in high grade abdominal lymphoma (patient 6) treated with intravenous combination chemotherapy are illustrated in Figure 5. By day 3 marked

**Table II** Pre-treatment $^{31}$P metabolite ratios and pH data

| | Pi/βATP[a] | PME/βATP | PDE/βATP | PME/Pi[b] | PDE/Pi | pH |
|---|---|---|---|---|---|---|
| Patient 1 | 0.34 | 1.34 | 0.95 | 4.00 | 2.83 | 7.23 |
| Patient 2 | 0.28 | 0.98 | 0.27 | 3.46 | 0.94 | 7.39 |
| Patient 3 | 0.18 | 1.38 | 0.37 | 7.70 | 2.09 | 7.39 |
| Patient 4 | 0.29 | 1.38 | 0.92 | 4.70 | 3.13 | 7.31 |
| Patient 5 | 0.27 | 2.01 | 2.10 | 7.51 | 7.86 | 7.16 |
| Patient 6 | 0.79 | 0.69 | 1.12 | 0.87 | 1.42 | 6.97 |
| Patient 7 | 1.87 | 1.86 | 1.14 | 0.99 | 0.61 | 7.24 |
| Patient 8 | 0.57 | 1.08 | 0.98 | 1.85 | 1.70 | 7.61 |

[a]$P = 0.036$, [b]$P = 0.018$ (Mann–Whitney U test); significance of differences between pre-treatment metabolite ratios of high and low grade NHL.

**Figure 2** Set of pre-treatment phosphorus spectra of low grade NHL obtained with the 1D-CSI technique from patient 5. 10 Hz line broadening has been applied. Peak assignments as shown (PME = phosphomonoesters; Pi = inorganic phosphate; PDE = phosphodiesters; PCr = phosphocreatine; α, β, γ – ATP = α, β, γ – adenosine trophosphate). Distance of each 1 cm slice of tissue off the surface coil from which spectra were obtained is also shown.

changes in Pi and PDE were already detectable, and were subsequently followed by a large reduction in tumour volume by day 7. Immediately before the next course of therapy relative metabolite levels had returned to near pre-treatment levels.

In patient 8 alterations in metabolites within 24 h of commencing therapy were detected with a relative increase in PDE and a decrease in βATP. This patient was studied very closely through the first three weeks of therapy with a multi-agent chemotherapeutic regime, PCOMMB (Philips, 1987). In this regime pairs of chemotherapeutic agents are given at weekly intervals and these studies allowed the response to each weekly drug combination to be assessed. A response to each drug combination was seen with a maximal rise in the PDE/βATP metabolite ratio by the second or third day

**Figure 3** Pre-treatment spectra from patients with (**a**) high grade NHL and (**b**) low grade NHL obtained 4 cm from the surface coil. Peak assignments as in Figure 2.

following each weekly treatment (Figure 6). A similar but less marked pattern of change was also seen in the Pi/βATP ratio. Immediately before the next treatment, metabolite levels were approaching pre-treatment baseline values.

Of the metabolite ratios examined the most consistent alterations with treatment were observed in the PDE/βATP ratio. All tumours showed marked increases in the PDE/βATP metabolite ratio (51–266%) with therapy. These

**Figure 4** Metabolite changes in low grade splenic lymphoma (patient 1), treated with oral alkylating agents. Results are shown as per cent change in metabolites in relation to pre-treatment value against days after commencing therapy. Timing of treatment, per cent reductions in tumour size, and alterations in tumour pH are also shown. -●- PME, -▲- Pi, -△- PDE, -◇- βATP.

**Figure 5** Metabolic changes of high grade abdominal lymphoma treated with combination chemotherapy (patient 6). Results are expressed as in Figure 4. -●- PME, -▲- Pi, -△- PDE, -◇- βATP.

**Figure 6** Serial studies in patient 8 showing the changes in the metabolite ratios PDE/βATP, and Pi/βATP over the first three weeks of treatment and their relation to the timing of therapy. Week 1 = mitozantrone 10 mg m$^{-2}$ i.v. and cyclophosphamide 350 mg m$^{-2}$ i.v.; week 2 = vincristine 1.4 mg m$^{-2}$ i.v., methotrexate 400 mg m$^{-2}$ over 4 h i.v.; week 3 as week 1; prednisolone 75 mg p.o. continuously. -▲- Pi/βATP, -●- PDE/βATP.

changes were seen before tumour shrinkage in all patients except patient 7. The maximum changes in this ratio and their relation to time after commencing chemotherapy are summarised in Figure 7.

Pre-treatment pH values are shown in Table II. pH values were normal or alkaline in low grade lymphoma (range 7.16–7.39 pH units). The range of pH values was greater in high grade NHL (6.99–7.61 pH units). No set pattern of pH change was seen with treatment, but pH remained very alkaline in some cases even in the presence of increased Pi (Figure 4).

### Discussion

The potential of MRS to assess treatment response in tumours has been well documented in animal models (Evanochko *et al.*, 1984; Sostman *et al.*, 1984; Maris & Chance, 1986; Daly & Cohen, 1989; Steen *et al.*, 1989). However, due to difficulties in performing *in vivo* MRS (low MR sensitivity of phosphorus containing compounds, low signal-to-noise ratio of *in vivo* spectra, poor spatial localisation of the MR signal, long examination time and patient compliance), only a few studies have been reported assessing early treatment response in human tumours (Ng *et al.*, 1987, 1989*a*; Semmler *et al.*, 1988*a*, *b*). These studies all used whole volume MRS localisation techniques therefore the spectra acquired from some of the tumours would have been contaminated with signal from surrounding non-tumour tissue. Phase encoding spectroscopic techniques have been used to assess treatment response in two patients with B cell non-Hodgkin's lymphoma of bone (Bryant *et al.*, 1988).

We have previously shown that the 1D-CSI technique is a reproducible method of obtaining localised *in vivo* $^{31}$P spectra from human organs (Glazer *et al.*, 1989; Smith *et al.*, 1990). It is important to note, however, that the metabolite ratios reported are relative ratios and are not absolute metabolite concentrations.

As lymphomas generally respond well to chemotherapy, and frequently present with very bulky superficial disease they are suitable to be studied by currently available MRS localisation techniques. This is the largest reported series of patients with similar tumour types studied with an image guided spectroscopic localisation technique to assess the $^{31}$P MRS characteristics that are associated with a response to chemotherapy.

Very reproducible spectral patterns were seen in both

**Figure 7** The maximum per cent change in the PDE/βATP metabolite ratio in relation to time after commencing therapy is shown for each patient. Patients numbered as in Table I (patient 7 not shown). -◆- Low grade NHL, -●- high grade NHL.

groups of lymphoma. The major pre-treatment difference in $^{31}$P characteristics between high and low grade lymphomas related to tumour bioenergetics. The Pi peak area was larger in relation to PME or βATP in high grade lymphoma. This presumably represents a larger hypoxic cell fraction in high grade lymphoma and may be due to out growth of tumour blood supply.

One may have expected the pH measured in some of these tumours to be more acidic due to lactate production but an alkaline pH is consistent with pH measurements reported for other human tumours (Ng *et al.*, 1989*b*; Oberhaensli *et al.*, 1986). Mechanisms proposed for the production of this alkaline tumour pH include raised intracellular pH in relation to cell growth and activation of $Na^+/H^+$ exchange (Schuldiner & Rozengurt, 1982; Hesketh *et al.*, 1985). The variation in pH in these lymphomas may reflect aspects of tumour heterogeneity, or have been related to limits of data resolution and the use of αATP as a reference from which to measure pH (Smith *et al.*, 1990).

Prominent PME resonances were seen in all tumours. The major constituents of the PME peak, phosphocholine and phosphoethanolamaine represent the anabolic pathway of phospholipid turnover and may relate to proliferation rates of tumours (Daly *et al.*, 1987). However, no clear relationship between relative PME peak area and grade of malignancy was seen. Further studies directed at the documentation of absolute concentrations of phosphomonoesters will be needed to clarify this issue.

$^{31}$P metabolite changes were observed before any reduction in tumour size had occurred. As the tumours studied were large and greatly exceeded the size of the surface coils used, we were confident that metabolite changes detected before reductions in tumour size reflected changes occurring in these tumours and were not due to partial volume effects.

In low grade NHL the metabolite changes observed with treatment consisted of increases in Pi and an associated fall in βATP, consistent with the development of an increasing proportion of hypoxic or dying cells within the tumour bulk. These changes were followed by relative increases in PDE. Glycerophosphorylcholine and glycerophosphorylethanolamine are the major constituents of the PDE peak and represent the catabolic side of phospholipid turnover (Daly *et al.*, 1987). Increased flux through this pathway as suggested by relative increases in PDE peak area may therefore represent mobilisation of cell membrane components associated with cell death.

In tumours with high growth fractions the response to chemotherapy can be rapid. In high grade lymphoma metabolite changes were detected by days 1 and 3 of chemotherapy (patients 8 and 6). Although similar changes in PDE and βATP were detected in high grade NHL as seen in low grade tumours, alterations in Pi in high grade NHL were more variable. This may relate to the timing of $^{31}$P studies after starting treatment, studies performed on day 1 or 2 in patient 6 may have detected earlier changes in tumour bioenergetics. The major difference in metabolic response to chemotherapy between high and low grade lymphoma was the time changes in the PDE/βATP ratio occurred in relation to the intensity of treatment given. In patients with low grade tumours given oral alkylating agents $^{31}$P MRS changes were seen between days 10 and 27 of starting therapy while in tumours given intensive combination chemotherapy changes were seen between days 1 and 5.

The serial studies in patient 8 allowed the response to weekly administrations of different chemotherapeutic drug combinations to be assessed, potentially allowing the more critical documentation of the efficacy of different agents in timed multiagent chemotherapy regimes.

All tumours responded well to their initial treatment schedules as measured by reduction of tumour bulk. The most consistent metabolic change seen in all tumours was an increase in the PDE/βATP ratio. Some tumours showed increases in the Pi/βATP ratio with treatment, although alterations in this ratio have been reported in untreated growth in animal tumours (Wehrle & Glickson, 1986) and in a human rhabdomyosarcoma unresponsive to therapy (Griffiths *et al.*, 1983). The PDE/βATP ratio may therefore be a more useful indicator of early response to chemotherapy in lymphomas, incorporating the metabolic changes seen in tumour bioenergetics with those of phospholipid turnover.

Increases in the PDE/βATP ratio have been reported as being associated with a good response to therapy in both subcutaneously implanted tumours in animals (Lutz *et al.*, 1989) and in human tumours (Semmler *et al.*, 1988*b*). *In vivo* MRS does not adequately resolve the individual resonances within the PME and PDE peaks but it has been suggested from high resolution tumour extract studies that it is an increase in the glycerophosphorylcholine component that is responsible for the relative increase in the PDE peak (Lutz & Wehrle, 1989).

In contrast to some recent reports we did not see any alterations in PME in the early studies after commencing therapy (Glaholm *et al.*, 1989; Ng *et al.*, 1989*a*). In later studies (approximately 5 months after initially starting treatment) in patients 1 and 4, marked decreases in PME were seen but these probably reflected alterations in the cell populations of the tumours as large reductions in tumour volume had by then occurred.

In this study, therefore, a reproducible slice selective MRS protocol was used to study a homogeneous group of patients. Very consistent metabolite changes were seen in these tumours after commencing chemotherapy. The PDE/βATP metabolite ratio may be a good metabolic indicator of early response to chemotherapy in lymphomas. These studies raise the possibility of performing specifically timed $^{31}$P MRS studies during treatment to assess the early efficacy of a treatment protocol in individual patients. The lack of appropriate metabolic change possibly being an indication for alteration in treatment at an earlier time than conventional practice currently allows. Obviously more detailed studies need to be performed in well defined patient groups before the role of $^{31}$P MRS in clinical oncology and therapeutics can be fully established, but these preliminary studies illustrate the feasibility and clinical potential of phosphorus MRS.

This work was supported by a grant from the North West Cancer Research Fund. We are grateful for the patients' co-operation during the course of these studies.

### References

BOTTOMLY, P.A. (1989). Human *in vivo* NMR spectroscopy in diagnostic medicine: clinical tool or research probe. *Radiology*, **170**, 1.

BRYANT, D.J., BYDDER, G.M. CASE, H.A. & 4 others (1988). Use of phosphorus-31 MR spectroscopy to monitor response to chemotherapy in non-Hodgkin's lymphoma. *J. Comput. Assist. Tomogr.*, **12**, 770.

COHEN, J.S., LYON, R., CHEN, C. & 5 others (1986). Differences in phosphate metabolite levels in drug-sensitive and -resistant human cancer cell lines determined by $^{31}$P magnetic resonance spectroscopy. *Cancer Res.*, **46**, 4087.

DALY, P.F. & COHEN, J.S. (1989). Magnetic resonance spectroscopy of tumours and potential *in vivo* clinical applications: a review. *Cancer Res.*, **49**, 770.

DALY, P.F., LYON, R.C., FAUSTINO, P.J. & COHEN, J.S. (1987). Phospholipid metabolism in cancer cells monitored by $^{31}$P NMR spectroscopy. *J. Biol. Chem.*, **262**, 14875.

EVANOCHKO, W.T., NG, T.C. & GLICKSON, J.D. (1984). Application of *in vivo* NMR spectroscopy to cancer. *Magn. Reson. Med.*, **1**, 508.

GLAHOLM, J., LEACH, M.O., COLLINS, D.J. & 5 others (1989). *In-vivo* $^{31}$P magnetic resonance spectroscopy for monitoring treatment response in breast cancer. *Lancet*, **i**, 1326.

GLAZER, G.M., SMITH, S.R., CHENEVERT, T.L., MARTIN, P.A., STEVENS, A.N. & EDWARDS, R.H.T. (1989). Image localised $^{31}$P magnetic resonance spectroscopy of the human liver. *NMR Biomed.*, **1**, 184.

GRIFFITHS, J.R., CADY, E., EDWARDS, R.H.T., MCCREADY, V.R., WILKIE, D.R. & WILTSHAW, E. (1983). $^{31}$P-NMR studies of a human tumour *in situ*. *Lancet*, **i**, 1435.

HESKETH, T.R., MOORE, J.P., MORRIS, J.D.H. & 4 others (1985). A common sequence of calcium and pH signals in the mitogenic stimulation of eukaryotic cells. *Nature*, **313**, 481.

LUTZ, N.W., LI, S.J., WEHRLE, W.P. & GLICKSON, J.D. (1989). Phospholipid metabolites in chemically treated RIF-1 tumours monitored by *in-vivo* $^{31}$P NMR spectroscopy. Proceedings of Society of Magnetic Resonance in Medicine, 8th Annual Meeting, 1, p. 398 (abstract).

LUTZ, N.W. & WEHRLE, J.P. (1989). Phospholipids and their metabolites in tumour extracts monitored by *in vitro* $^{31}$P NMR spectroscopy. Proceedings of the Society of Magnetic Resonance in Medicine, 8th Annual Meeting, Works in Progress, 1074 (abstract).

MARIS, J.M. & CHANCE, B. (1986). Magnetic resonance spectroscopy of neoplasms. In *Magnetic Resonance Annual*, Kressel, H.Y. (ed.) p. 213. Raven Press: New York.

NARUSE, S., HIRAKAWA, S., HORIKAWA, Y. & 5 others (1985). Measurements of *in-vivo* $^{31}$P nuclear magnetic resonance spectra in neuroectodermal tumours for the evaluation of the effects of chemotherapy. *Cancer Res.*, **45**, 2429.

NG, T.C., VIJAYAKUMAR, S., MAJORS, A.W., THOMAS, F.J., MEANEY, T. & BALDWIN, N.J. (1987). Response of a non-Hodgkin lymphoma to $^{60}$Co therapy monitored by $^{31}$P MRS *in situ*. *Int. J. Radiat. Oncol. Biol. Phys.*, **13**, 1545.

NG, T.C., GRUNDFEST, S., VIJAYAKUMAR, S. & 7 others (1989*a*). Therapeutic response of breast carcinoma monitored by $^{31}$P MRS *in situ*. *Magn. Reson. Med.*, **10**, 125.

NG, T.C., MAJORS, A.W., VIJAYAKUMAR, S. & 8 others (1989*b*). Human neoplasm pH and response to radiation therapy: P-31 MR spectroscopy studies *in situ*. *Radiology*, **170**, 875.

OBERHAENSLI, R.D., HILTON-JONES, D., BORE, P.J., HANDS, L.J., RAMPLING, R.P. & RADDA, G.K. (1986). Biochemical investigation of human tumours *in vivo* with phosphorus-31 magnetic resonance spectroscopy. *Lancet*, **ii**, 8.

PHILIPS, J.K. (1987). Mitozantrone combinations in NHL and Hodgkin's disease. In *Proceedings 3rd United Kingdom Novantrone Symposium*, Hatt, K.J. (ed.) p. 51. Wiley: London.

SCHULDINER, S. & ROZENGURT, E. (1982). $Na^+/H^+$ antiport in Swiss 3T3 cells: mitogenic stimulation leads to cytoplasmic alkalinisation. *Proc. Natl Acad. Sci. USA*, **79**, 7778.

SEMMLER, W., GADEMANN, G., BACHERT-BAUMANN, P., ZABEL, H.J., LORENZ, W.J. & VON KAICK, G. (1988*a*). Monitoring human tumour response to therapy by means of P-31 MR spectroscopy. *Radiology*, **166**, 533.

SEMMLER, W., GADEMANN, G., SCHLAG, P. & 4 others (1988*b*). Impact of hyperthermic regional perfusion therapy on cell metabolism of malignant melanoma monitored by $^{31}$P MR spectroscopy. *Magn. Reson. Imaging*, **6**, 335.

SMITH, S.R., MARTIN, P.A., DAVIES, J.M. & EDWARDS, R.H.T. (1990). Characterisation of the spleen by *in-vivo* image guided $^{31}$P magnetic resonance spectroscopy. *NMR Biomed.* (in the press).

SOSTMAN, H.D., ARMITAGE, I.M. & FISCHER, J.J. (1984). NMR in cancer. High resolution spectroscopy of tumours. *Magn. Reson. Imaging*, **2**, 265.

STEEN, R.G. (1989). Response of solid tumours to chemotherapy monitored by *in-vivo* $^{31}$P magnetic resonance spectroscopy: a review. *Cancer Res.*, **49**, 4075.

TAYLOR, D.J., BORE, P., STYLES, P., GADIAN, D.G. & RADDA, G.K. (1983). Bioenergetics of intact human muscle. A $^{31}$P nuclear magnetic resonance study. *Mol. Biol. Med.*, **1**, 77.

WEHRLE, J.P. & GLICKSON, J.D. (1986). $^{31}$P NMR spectroscopy of tumours *in vivo*. *Cancer Biochem. Biophys.*, **8**, 157.